# A Systematic Review on the Effectiveness of Pre-Harvest Meat Safety Interventions in Pig Herds to Control *Salmonella* and Other Foodborne Pathogens

**DOI:** 10.3390/microorganisms9091825

**Published:** 2021-08-27

**Authors:** Maria Rodrigues da Costa, Joana Pessoa, Diana Meemken, Truls Nesbakken

**Affiliations:** 1 Epidemiology Research Unit, Department of Veterinary and Animal Science, Northern Faculty, Scotland’s Rural College (SRUC), An Lòchran, 10 Inverness Campus, Inverness IV2 5NA, Scotland, UK; maria.costa@sruc.ac.uk; 2 Pig Development Department, Teagasc, Animal and Grassland Research and Innovation Centre, Moorepark, Fermoy, P61 C997 Co. Cork, Ireland; joana.pessoa@teagasc.ie; 3 Section of Herd Health and Animal Husbandry, School of Veterinary Medicine, University College Dublin, Belfield, 4 Dublin, Ireland; 4 M3-BIORES-Measure, Model & Manage Bioresponses, KU Leuven, Kasteelpark Arenberg 30, 3001 Leuven, Belgium; 5 Institute of Food Hygiene and Food Safety, Freie Universität Berlin, Working Group Meat Hygiene, Königsweg 67, D-14163 Berlin, Germany; 6 Faculty of Veterinary Medicine, Department of Production Animal Clinical Sciences, Norwegian University of Life Sciences, P.O. Box 5003, 1432 Ås, Norway; Truls.Nesbakken@nmbu.no

**Keywords:** farm practices, foodborne pathogens, pig production, pork, pre-harvest interventions, salmonella, zoonoses

## Abstract

This systematic review aimed to assess the effectiveness of pre-harvest interventions to control the main foodborne pathogens in pork in the European Union. A total of 1180 studies were retrieved from PubMed^®^ and Web of Science for 15 pathogens identified as relevant in EFSA’s scientific opinion on the public health hazards related to pork (2011). The study selection focused on controlled studies where a cause–effect could be attributed to the interventions tested, and their effectiveness could be inferred. Altogether, 52 studies published from 1983 to 2020 regarding *Campylobacter* spp., *Clostridium perfringens*, Methicillin-resistant *Staphylococcus aureus*, *Mycobacterium avium*, and *Salmonella* spp. were retained and analysed. Research was mostly focused on *Salmonella* (*n* = 43 studies). In-feed and/or water treatments, and vaccination were the most tested interventions and were, overall, successful. However, the previously agreed criteria for this systematic review excluded other effective interventions to control *Salmonella* and other pathogens, like *Yersinia enterocolitica*, which is one of the most relevant biological hazards in pork. Examples of such successful interventions are the Specific Pathogen Free herd principle, stamping out and repopulating with disease-free animals. Research on other pathogens (i.e., Hepatitis E, *Trichinella spiralis* and *Toxoplasma gondii*) was scarce, with publications focusing on epidemiology, risk factors and/or observational studies. Overall, high herd health coupled with good management and biosecurity were effective to control or prevent most foodborne pathogens in pork at the pre-harvest level.

## 1. Introduction

In Europe, the current most important foodborne hazards in pork include microbiological agents (for example, *Salmonella*) [1,2]. The new risk-based meat inspection legislation was laid out in agreement with these hazards and proposes a risk-informed visual-only inspection where the focus is on the prevention and control of meat-borne hazards before slaughter, such as on-farm or at transport [3]. This integration of measures along the food chain requires cooperation between the different stakeholders and has the potential to consistently reduce the risks associated with meat-borne hazards. 

In pig production, key concepts for interventions at the herd level are the control of the purchase and flow of animals, in particular, at the top of the breeding pyramid, the control of feed, internal and external biosecurity, and the categorisation of herds that are carriers of specific pathogens. Interventions at the herd level may also contribute to a more sustainable and “clean” production, while also solving general problems connected to the environment by avoiding recycling of zoonotic hazards like *Salmonella* at the farm level [3]. Many of these control measures are described in the literature but their effectiveness to control the different foodborne pathogens related to pork has not been addressed. This work aimed to collate and synthesize evidence on the effectiveness of pre-harvest interventions to control foodborne pathogens in pork. 

The foodborne hazards targeted in this systematic review were based on the European Food Safety Authority [EFSA] scientific opinion on the public health hazards to be covered by inspection of pork [1]. This scientific opinion collated a list of relevant biological hazards for which there is evidence (in the literature and/or in data provided by Member States) that they occur or may occur in pigs in Europe and that can be transmitted via food to humans. Fifteen biological hazards were selected. Within these, *Salmonella* was considered of high relevance in the EU, while *Toxoplasma* *gondii, Trichinella* spp. *and Yersinia enterocolitica*, were considered of medium relevance. The control and prevention measures, especially pre-harvest interventions, indicated for *Salmonella* spp. and *Y. enterocolitica* would be beneficial for controlling other microbial hazards [1]. Other hazards such as *Campylobacter* spp., *Clostridium botulinum, Clostridioides difficile, Clostridium perfringens,* Hepatitis E virus, *Listeria monocytogenes*, *Mycobacterium* spp., *Sarcocystis suihominis*, *Staphylococcus aureus* (including Methicillin Resistant *Staphylococcus Aureus* (MRSA)), *Taenia solium cysticercus*, and Verotoxinogenic-producing *Escherichia coli* were considered of low relevance, but likely to be present based on the frequency of detection of hazards in pork carcasses after chilling, and so were equally included in this systematic review. 

## 2. Materials and Methods

This systematic review is part of a set of three reviews on the effectiveness of pre-harvest interventions to control foodborne pathogens in broilers, pigs, and bovine. Such work was framed in the context of the RIBMINS Cost Action (please refer to the Acknowledgements section). Likewise, the methods followed are similar to those described by Pessoa et al. [4] and the work presented here was conducted by the same two review coordinators, and two volunteer researchers. The backbone of the methodology used followed the PRISMA (Preferred Reporting Items for Systematic Reviews and Meta-Analyses) statement [5] and EFSA’s guidelines for conducting systematic reviews for food and feed safety assessments [6]. PRIMA’s checklist for systematic reviews has been completed and is available as Supplementary Material (S1).

All literature searches were conducted on two online databases (PubMed^®^ and Web of Science) on 8 February 2021. Only peer-reviewed studies written in English and published before 31 December 2020 on the effectiveness of pre-harvest meat safety interventions to control 15 foodborne pathogens (those highlighted by EFSA [1]) in pigs were included. Searches were restricted to title and abstract. 

**Figure 1:**
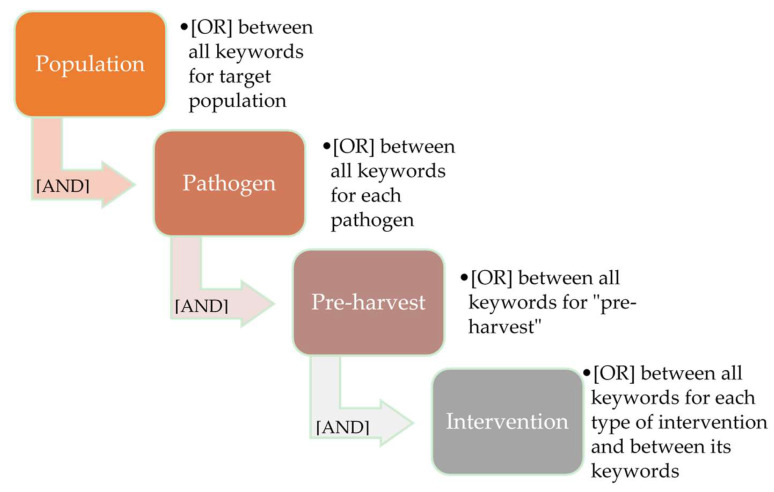
Structure outline of text strings used for the searches conducted in PubMed^®^ and the Web of Science databases on 8 February 2021 (reproduced from Pessoa et al. [4], which employed the same methods). The search strings used are available as Supplementary Material.

Figure 1 shows the composition of the search strings used in PubMed^®^ and in Web of Science. Keywords and search strings specific to each pathogen are presented in Table 1. The detailed search strings employed in each database are available as Supplementary Material (S2).

EndNote was used to import all search results. All duplicates were removed. The set of inclusion and exclusion criteria to filter titles and abstracts is presented in Table 2. One of these criteria was to only select scientific papers with experimental/controlled study designs. This decision was made to highlight the presumptive causal effect of the interventions tested. One co-author screened all 1180 records using these criteria and selected 87 papers for further analysis. After that, the selected papers were retrieved and two co-authors (in parallel and blinded to each other’s decisions) read the full texts using the same eligibility criteria (Table 2). Exclusion of records had to be agreed by both co-authors. Records upon which agreement was not reached were reviewed by a third co-author, producing a final decision. Table 1 shows the list of records included in all stages of the systematic review process.

The data within the final 52 records included in this work were extracted onto a database (stored in a Microsoft Office Excel^®^ spreadsheet). Studies (i.e., any peer-reviewed original research in which the authors collected, analysed, and reported their own data) were documented and classified based on the pre-harvest intervention. Other information (country of study, year it took place, type of experiment, subject type, number of experimental units, sample type, outcome measured, and estimate of effectiveness) was also retrieved. Some studies assessed the efficacy of multiple interventions. For *Salmonella* studies, the comparison of each treatment (intervention) with the control was recorded as a trial and, if possible, detailed information was collected for each trial. For each *Salmonella*-related study, the results of the interventions tested were summarised according to whether there was a reduction of *Salmonella* shedding, reduction of *Salmonella* counts or improvement of protective immunity. Whenever the outcome of an intervention was measured through several time-points, data collected at the end of the study (i.e., closer to the slaughter date) were preferred.

## 3. Results

A total of 1180 unique studies published between 1968 and 2020 were retrieved through the search strings run on PubMed^®^ and Web of Science for the 15 pathogens included in this systematic review. After the review process described, a final list of 52 studies published between 1983 and 2020 were retained. This list is available as Supplementary Material (S3). In the full text analysis and selection, the authors had an agreement rate of 95.4% (83/87). Four studies were reviewed by a third author to decide upon its selection. Due to the decision of the third reviewer, one study out of the four was retained in the systematic review. All authors were blinded to each other’s final decisions. The list of studies excluded during the full-text evaluation (*n* = 35) is available as Supplementary Material (S4). Only five of the pathogens listed had studies meeting the defined criteria (Table 1).

### 3.1. Campylobacter

The two studies retained for *Campylobacter* spp. tested the efficacy of probiotics to reduce the colonisation of this pathogen as competitive exclusion and consequently reduce the risk of carcass contamination during slaughter. Bratz et al. [7] tested the inhibitory activity of the strain *E. faecium* NCIMB 10,415 against *C. coli* in vivo. This probiotic was administered as a diet supplementation in sows (three weeks before parturition) and to their progeny from 12 days of age until the end of the trial. Sows and piglets from the control group were not fed any supplements. The authors reported that all piglets were already naturally colonised with *C. coli* before the challenge trial, which was a unique dosage of 7 × 10^7^ cfu strain *C. coli* 5981 via an intragastric application. The excretion load of *C. coli* was monitored for 28 days and the results indicate that the tested probiotic did not significantly affect *C. coli* excretion levels in pigs. In the other *Campylobacter* spp.-related study, Hasan et al. [8] tested the effects of diet supplementation of resin acid-enriched composition (RAC) in the last week of gestation on colostrum yield, composition and gut microbiota. Three trials in three different commercial herds were performed. Apart from the colostrum yield and composition improvements, the diet supplementation with RAC seemed to shift the relative abundance of opportunistic and pathogenic agents, such as *Campylobacter*, potentially reducing the risk of piglet infection.

### 3.2. Clostridium Perfringens

Five studies assessing the efficacy of vaccinations (*n* = 4) and probiotics (*n* = 1) were retained. Of the vaccination studies, two of them tested sow and gilt vaccination strategies to control necrotizing enteritis (*C. perfringens* type C; [9]) and *C. perfringens* type A-associated diarrhoea in piglets [10]. Two other studies assessed the efficacy of piglet vaccination to control neonatal diarrhoea caused by *Clostridioides difficile* [11] and necrotizing enteritis (*C. perfringens* type C; [12]). 

One study assessed the efficacy of competitive exclusion by administering a probiotic to control diarrhoea in piglets [13]. The cocktail tested contained living strains of attenuated *C. perfringens* type A and non-pathogenic *Escherichia coli* and it was administered *per os* to newborn piglets in a commercial farm with a history of neonatal diarrhoea caused by *C. perfringens* type A. 

All studies reported positive outcomes for the interventions tested. Two studies reported a reduction in piglet mortality, which corresponded to a numerical but not statistical reduction in the study by Kelneric et al. [9] and to a statistically significant mortality rate reduction in the study by Unterweger et al. [13]. Hammer et al. [10] documented an increase of neutralizing antitoxins against *C. perfringens* type A in piglets born from vaccinated dams compared to those born of dams not vaccinated, and Richard et al. [12] reported higher titres against *C. perfringens* type C in vaccinated piglets when compared to those not vaccinated. Finally, in the study by Oliveira et al. [11], the authors documented a reduction of the isolation of *C. perfringens* in diarrhoea samples after administering a non-toxigenic strain of *C. difficile* to one-day-old piglets on a commercial pig farm.

### 3.3. Methicillin-Resistant Staphylococcus Aureus (MRSA) 

Only one study on MRSA met the inclusion criteria. This study reported the results of a randomised control trial to test the efficacy of a thorough cleaning disinfection protocol for sows and the environment (farrowing house and nursery unit) to reduce the prevalence of livestock-associated MRSA in sows and their progeny [14]. Two farrow-to-finish commercial farms with a 3-week batch system were enrolled in the study, and, in each farm, six sow batches were tested (three batches tested and three batches as control, all with approximately 20 sows). Results showed that the tested disinfection protocol reduced temporarily the sow and piglet MRSA status, but it did not equate to a final reduction in MRSA at weaning or in the nursery unit. 

### 3.4. Mycobacterium Avium Complex

Hines et al. [15] tested the efficacy of vaccination for *Mycobacterium avium* with two different vaccines in preventing infection and disease in experimentally challenged pigs. The study tested a killed “whole cell” *M. avium* serovar 2 as a vaccine, and a conjugated MIF-A3 subunit vaccine. The results showed that the killed vaccine did not prevent infection but attenuated its severity with regard to gross and macroscopic lesions, when compared to the pigs vaccinated with the subunit vaccine. The latter did not prevent infection and the lesions observed were very similar to pigs vaccinated with a sham vaccine (saline solution). 

### 3.5. Salmonella 

In total, 43 studies testing different pre-harvest interventions for the control of *Salmonella* infections in pigs were found [16,17,18,19,20,21,22,23,24,25,26,27,28,29,30,31,32,33,34,35,36,37,38,39,40,41,42,43,44,45,46,47,48,49,50,51,52,53,54,55,56,57,58]. Table 3 compiles a description of the studies retained, with a summary of the trials reported in them and their results. Forty-one studies were designed to investigate on-farm interventions, and four studies tested transport interventions [19,20,29,48]. 

In total, 86 trials were identified among the 43 *Salmonella* studies selected. The most tested type of intervention was in-feed and/or water treatments. Out of the 32 trials that tested different acids in-feed or water (i.e., sorbic acid, sodium butyrate, or blends of citric acid, formic acid and essential oils) and other feed-related interventions like fermentation or herbal extracts, including prebiotics, 23 (72%), reported positive results. 

Most vaccination trials (88%, 21/24) reported positive results. The most common challenges reported were the lack of cross-protection of some vaccines against other serotypes and the potential interference of vaccination-induced antibodies in the meat juice sampling for *Salmonella* control purposes at slaughter. The three trials reporting vaccination interventions without positive results were: (1) a trial where an oral vaccine administered to piglets at 3 weeks of age lowered transmission (numerically) but failed to reduce excretion of *Salmonella* Typhimurium [36]; (2) a trial where the vaccination of sows with a commercial vaccine to control of *Salmonella* Typhimurium infections failed to decrease the prevalence of *Salmonella* Typhimurium field strain positive lymph nodes at slaughter in finisher pigs born to those sows [58]; and (3) a trial where a commercial oral vaccine based on *Salmonella serovar* Choleraesuis variety Kurzendorf was administered between 24 and 72 h after birth and was not effective in reducing the within-herd spread of *Salmonella* during the finishing phase or the frequency of carcass contamination at slaughter, with *Salmonella* Typhimurium being isolated from lymph nodes of vaccinated pigs [57].

Eight trials tested the efficacy of administering antimicrobials to control *Salmonella* infections [16,24,27,39,55]. The only trial with a positive result was reported in a study combining intramuscular administration of ceftiofur with off-site early weaning at 10–15 days of age, where Nietfeld et al. [18] concluded that this intervention prevented *Salmonella* spp. infection in grow-finish pigs. 

Of the seven trials reporting results on the efficacy of cleaning and disinfection interventions, two exclusively tested it on transport [19,29], and only one other trial reported the effect of cleaning and disinfection on-farm alone [50]. Rajkowski et al. [19] tested the effect of washing and sanitizing lorries after each load and it significantly reduced levels of *Salmonella* detected on lorries. Similarly, Mannion et al. [29], who tested bacterial loads and isolates on lorries carrying pigs from high- and low-risk farms during and after transport, and also after washing, commented on the need for better cleaning of lorries after each transport (or load), especially when transporting pigs from “high-risk herds of *Salmonella*”. The authors found isolates identical to those on farm on lorries after washing. Finally, Martelli et al. [50] tested the application of a rigorous disinfection protocol of finisher facilities on-farm with the objective of eliminating *Salmonella*, comparing it to the normal procedures followed by farmers. The authors found that this protocol significantly reduced the prevalence of *Salmonella* in pigs prior to slaughter. Other studies reporting trials testing cleaning and disinfection have been described before or did not obtain positive results [17,24].

Several trials investigated the efficacy of a combination of different interventions. These included a combination of cleaning and disinfection with off-site early weaning [17]; feed withdrawal and duration of transport [20]; a combination of chlorate treatment and topical disinfection administered to piglets together with early weaning [28]; and a combination of different particle size and acids in feed [32]. All of the cited studies reported positive results in one or more of the interventions tested. Four trials (within three studies) testing combined interventions did not report positive effects [24,27,32]. 

Nineteen trials tested other types of interventions, either alone or in combination with the intervention types described above. Examples of other interventions tested are off-site early weaning [17,18], washing and disinfecting of lorries [19,29], split marketing [31], different space allowances [27,40], and feed withdrawal and transport times [20,48]. 

## 4. Discussion

Over the years, several studies have been published on pre-harvest interventions to control foodborne zoonoses in pork. In this systematic review, we aimed to identify controlled studies that could provide a certain level of confidence regarding the effectiveness of the interventions tested, rather than identifying risk factors for the control of infections by the biological hazards listed. The papers selected for full text analysis were published over an extended timeframe (1983 to 2020). Across these years, *Salmonella* was one of the biggest concerns, with related publications representing 49% (785/1606) of the initial search returns. This is a direct consequence of its high relevance as a biological hazard of concern in pork meat (EFSA, 2011) and of the vast research undertaken to address this issue. Indeed, *Salmonella* is currently the second most reported foodborne pathogen in the EU, having been the most reported pathogen for many years, and is commonly associated with the consumption of pork [59]. The fact that few studies were retrieved for other pathogens within the criteria defined highlights the need for further research on the effectiveness of pre-harvest interventions to control these hazards. Another possible explanation is that some of these pathogens may be more cost-efficiently controlled by post-harvest interventions.

### 4.1. Salmonella

*S.* Typhimurium is the most common *Salmonella* in pig herds in most European countries, and this agent is known to be introduced into the herds by healthy carriers among the breeding animals and also by contaminated feed [60]. However, there is an extensive list of additional risk factors connected to biosecurity that should be tackled at the herd level, such as birds, rodents, insects, water, manure, humans entering the piggery and environment, etc. [61]. Unsurprisingly, several types of interventions to control *Salmonella* were found in the literature. In line with the risk factors identified in the literature for *Salmonella* infections, the most common pre-harvest interventions identified were in-feed and/or water treatments as well as vaccination. Among the most effective interventions, cleaning and disinfection and vaccination appeared to have high success rates. Nevertheless, across all trials, the results for *Salmonella* are very encouraging, with 76% (65/86) of the trials assessed reporting positive results. Although there is no scope in this paper to debate the reasons for intervention failure, including vaccination failure, reported in the studies evaluated, other studies have systematically assessed the effect of vaccination as a control strategy against *Salmonella* infection in pigs [62], the efficacy and quality of evidence for five on-farm interventions for *Salmonella* reduction in grow-finish swine [63], and the evidence for effectiveness of primary production interventions to control *Salmonella* in pork [64]. In spite of these positive results and vast literature published, the endemic *Salmonella* spp. infections in pig herds across the world reflect how challenging it is to control this pathogen. 

At national level, Finland, Norway and Sweden have documented that the successful control of *Salmonella* in cattle, pigs and poultry through pre-harvest interventions is possible. Heat-treatment of feed, and starting with breeding animals free from *Salmonella* at the top of the breeding pyramid have probably been the most important measures [61]. The food safety authorities have an important role following up positive herds to prevent transmission to other herds, humans and food, by prohibiting the purchase and transportation of animals and foods from infected farms. This highlights that prevention rather than control is a feasible pre-harvest intervention when targeting this hazard in pork.

### 4.2. Other Pathogens

#### 4.2.1. *Campylobacter*

Multiple studies have shown that pigs are an important reservoir of *C. coli* and that it is difficult to control this species at the herd level [65,66,67]. It seems more cost-efficient to control this agent post-harvest. Given the sensitivity of *Campylobacter* to both freezing and drying, blast chilling has proved to significantly reduce this agent on carcasses’ surface [68]. Even after traditional slow chilling there is a significant decline of this agent [69]. Accordingly, pig carcasses and pork are not regarded as an important source of *Campylobacter* in a public health context as confirmed by most epidemiological studies [70,71]. According to Roux et al. [72], “The aetiology of human *C. coli* infections is similar in a number of respects to *C. jejuni* but there are important differences. There is an increased risk of *C. coli* infection in the older people, in people who live in rural areas and during the summer months. Public health together with national and international food safety agencies should take these differences into account when considering interventions to reduce the incidence of this gastrointestinal pathogen”.

#### 4.2.2. *C. perfringens*

All of the *C. perfringens*-related studies reported outcomes referring to the control of disease in piglets and none reported or discussed the possible effects of the tested intervention to control shedding of this pathogen in the faeces. Thus, in spite of the apparent efficacy of the pre-harvest interventions tested, such as vaccination and competitive exclusion, these were not meant to control the risk of foodborne infections by *C. perfringens* acquired by pork consumption. This is likely to be related to the low risk this pathogen represents since the “risk of disease seems not to be correlated with occurrence in raw meat but rather to improper hygiene and storage” [1], meaning that this pathogen is mostly controlled by post-harvest interventions.

#### 4.2.3. MRSA

The tested disinfection protocol in one study temporarily reduced the sow and piglet MRSA status, but it did not equate to a final reduction in MRSA at weaning or in the nursery unit. 

Other similar trials testing thorough cleaning and disinfection of the facilities or sow washing and disinfection were captured in this review, but the absence of control groups dictated their exclusion. 

However, more comprehensive measures have been successful. Norway has established a unique control strategy for MRSA in their pig population, which includes population-wide annual surveillance, in addition to contact tracing upon detection of MRSA in pig farms and farm workers. Restrictions prohibit trade of live pigs carrying MRSA, other than directly to slaughter. Following depopulation, the farm owner is responsible for thorough washing and disinfection of farm premises. After a mandatory down-time, the farm is repopulated with pigs from MRSA-negative herds [73]. The surveillance programme in 2019 detected only one pig herd with MRSA. In total, 722 herds were included in the survey [74].

#### 4.2.4. *Mycobacterium avium*

The authors of the study [15] reported that it was not possible to determine if the vaccine tested had significantly reduced the bacterial load of the animals challenged, since low numbers of organisms were cultured. More importantly, the authors also note that the vaccines were not effective in controlling the foodborne zoonotic potential of *M. avium* given that the elimination of the organism was not achieved.

#### 4.2.5. Hepatitis E Virus

One unexpected result was the absence of Hepatitis E virus-related papers retained for analysis, even after a relatively high number of papers were detected in the initial search (*n* = 77). This pathogen has been earning attention in the last few years. However, none of these papers fulfilled the criteria for inclusion in this systematic review. According to Meester et al. [75], pigs are the main reservoir of the HEV (genotypes 3 and 4) worldwide, and humans can become infected by consumption of pork or contact with pigs. As HEV is persistently present on most pig farms, current risk mitigation strategies should focus on lowering transmission within farms, especially between farm compartments. Vaccination of pigs may aid HEV control in the future [76].

#### 4.2.6. *Y. enterocolitica*

Due to the exclusion criteria, studies on *Y. enterocolitica* were not retained. However, the risk assessment by EFSA [1] identified *Y. enterocolitica* as one of the most relevant biological hazards in the context of meat inspection of swine. Accordingly, this agent should be covered by preventive measures in the meat chain. At the farm level, some risk factors have been identified as contributors for seropositive herds, namely:

1. Buying animals from herds with an unknown carrier state for human pathogenic *Y. enterocolitica* [77,78];
2. Buying piglets from more than one farm [78,79,80]; and
3. Use of non-municipal water sources and having a continuous production (instead of applying an all-in/all-out strategy) [79].

One study indicated that clusters (health and breeding pyramids) of pig herds free from animal diseases (Specific Pathogen Free (SPF) herds) also seem to be free from *Y. enterocolitica* [81]. Some of these SPF herds were even free from *Campylobacter* spp. [82].

However, there are several control options at the slaughterhouse [83]. However, after slaughter, control measures seem ineffective, since *Y. enterocolitica* can survive and grow during cold storage and under modified atmospheres [83,84].

#### 4.2.7. *T. gondii*

The risk assessment by EFSA [1] also identified *T. gondii* as one of the most relevant biological hazards in the context of meat inspection of swine, but no studies of *T. gondii* were retained. Former studies show that the prevalence of *T. gondii* in pigs has decreased considerably in areas with intensive farm management [85,86]. However, pork originating from outdoor pig husbandry systems including those that are more welfare friendly such as free roaming, poses a higher risk compared to the indoor system [87], and this was not the focus of this systematic review. Other interesting approaches to interrupt the zoonotic circle of *T. gondii* are the vaccination of cats [88] or the control of the cat population in endemic regions [89]. So far, no commercial vaccine for cats is available.

### 4.3. Limitations of This Review

The results of this review and the implications inferred from them are valid within the context of the inclusion and exclusion criteria as defined *a priori*. This means that papers which did not contain a control group and from which a causal effect of the intervention tested could not be inferred were rejected. This decision was made to minimize bias and to eliminate confounding factors. However, observational studies are prevalent in the literature and the quality of the evidence provided by some of these studies should be graded up, provided that their results are robust. For example, identifying a strong correlation between high biosecurity and cleaning standards and low *Salmonella* shedding across multiple farms is a strong indication that such interventions are likely to be effective under the various scenarios of each farm. Conversely, randomized control trials reporting positive effects (*P* < 0.05) rarely declare the magnitude of this effect (i.e., adjusted R-squared with the proportion of the variability explained by the factor tested in the outcome variable). Mapping and summarizing the risk factors for each foodborne pathogen and the pre-harvest interventions proposed to tackle them is a task yet to be undertaken.

## 5. Conclusions

Some foodborne pathogens appear to be best controlled at a post-harvest level. However, overall, high herd health status coupled with good management and biosecurity were effective to control or prevent most foodborne pathogens in pork at the pre-harvest level. In spite of not having been included in the review, the SPF herd principle, stamping out and repopulating with disease-free animals, has been reported as a feasible and effective intervention to control foodborne pathogens like *Salmonella*, *Y. enterocolitica* and MRSA.

## Figures and Tables

**Table 1:** Flow of information through the systematic review for 15 foodborne pathogens, including keyword and/or string searched for each pathogen.

Pathogen	Keyword and/or String Searched	Records Identified	Records after Duplicates’ Removal	Records Retained after Abstract Screening	Records Retained after Full Text Screening
*Clostridium botulinum*	clostridium botulinum OR botulism	3	3	0	0
*Clostridioides difficile*	clostridium difficile OR c. difficile OR clostridioides difficile	8	7	0	0
*Clostridium perfringens*	clostridium perfringens OR c. perfringens OR clostridial diarrh *	43	33	9	5
*Campylobacter* spp.	Campylobacter * OR “Campylobacter jejuni” OR “campylobacter coli”	156	115	3	2
Herpes virus type E	hepatitis E OR hepE	101	77	0	0
*Listeria monocytogenes*	listeria monocytogenes OR listeriosis	12	11	0	0
MRSA	methicillin resistant staphylococcus aureus OR MRSA OR resistant s.aureus	194	139	9	1
*Mycobacterium avium complex*	mycobacterium OR tuberculosis	27	23	3	1
*Salmonella* spp.	Salmone *	785	555	57	43
*Sarcocystis* spp.	sarcocystis	9	7	0	0
*Taenia solium*	taenia solium cysticercus OR cysticercosis OR taeniasis	12	12	0	0
*Toxoplasma gondii*	toxoplasma gondii OR toxoplasmosis	101	77	2	0
*Trichinella spiralis*	Trichin *	63	50	2	0
VTEC	VTEC OR verotoxigenic *E. coli* OR verotoxigenic escherichia coli OR verocytotoxigenic *E. coli* OR shiga toxin-producing *E. coli*	5	5	1	0
*Yersinia enterocolitica*	Yersini *	87	66	1	0
TOTAL		1606	1180	87	52

Legend: MRSA—Methicillin-resistant *Staphylococcus aureus* (MRSA); VTEC—Verocytotoxin-producing *Escherichia coli* (VTEC). The “*” corresponds to the code/character used for the searches in the online databases. By using a * we indicate that the search motor should retrieve all words that start like the example given, regardeless of how they end.

**Table 2:** Eligibility (inclusion and exclusion) criteria used for the screening of title/abstracts and full texts. Reproduced and adapted from Pessoa et al. [4], where the same set of exclusion and inclusion criteria were used.

PICO ^1^	Inclusion Criteria	Exclusion Criteria
Population	Animal species being evaluated: must include (but not limited to) pigs	Does not include actual or theoretical <pathogen> infection/contamination in pigs
	Unit of study [animal, herd, house, barn, farm] and [surfaces, food, water, environment, drinkers, feeder, other animals]	Others
Intervention	Interventions to control/reduce/eradicate <pathogen> in pigs	Studies not mentioning control/reduce/eradicate interventions for < pathogen> in pigs
	Interventions on-farm or during transport (pre-harvest)	Interventions on lairage, at slaughter and post-harvest
	Field/experimental studies	Lab/bench studies
Comparison	Control group present [group subjected to no intervention]	Control group absent
Outcomes	Provides some measure of the efficacy of the intervention	Efficacy of the intervention not measured
Others	Language: English	Other languages
	Peer-reviews	Grey literature

^1^ PICO (participants, interventions, comparisons, and outcome(s))-framework to formulate research questions, following the methods proposed in the PRISMA statement [5].

**Table 3:** Descriptive characteristics of the 86 trials described in the 43 studies investigating pre-harvest interventions to control *Salmonella* spp. in pork.

Variable	Category	*Salmonella* Studies and Trials
		43 studies, n (%)
Location of intervention	On-farm Transport	41 (95.3) 4 (9.3) ^1^
Study setting	Commercial farm Research farm	34 (79.1) 10 (23.2) ^2^
		86 trials, n (%)
Type of intervention ^3^	Cleaning & disinfection Combination of measures Feed and/or water treatments - Acids in water - Acids in feed - Other (i.e., fermentation) Antibiotics Vaccination Other ^4^	7 (8.1); positive results *: *n* = 6 19 (22.1); positive results *: *n* = 15 32 (37.2); positive results *: *n* = 23 4 (12.5) 21 (65.6) 9 (28.1) 8 (9.3); positive results *: *n* = 1 24 (27.9); positive results *: *n* = 21 19 (22.1); positive results *: *n* = 11

^1^ Two studies tested both on-farm and transport interventions. ^2^ One study had two trials, one performed in a commercial farm setting, and another performed under controlled research laboratory conditions. ^3^ The comparison of each treatment (intervention) with the control was recorded as a trial. Some trials consisted of a combination of approaches (i.e., acids in-feed and in water simultaneously). The trials are repeated across different categories if they fit in more than one type of intervention. ^4^ Examples of other interventions tested are off-site early weaning, washing and disinfecting of lorries, split marketing approaches, and different space allowances. * Trials which reported at least one positive result (i.e., reduction of *Salmonella* shedding, increase of protective immunity).

## Data Availability

The data generated and analysed during this study are included in this published article (and its Supplementary Information Files).

## Supplementary Materials

The following are available online at https://www.mdpi.com/article/10.3390/microorganisms9091825/s1, S1: PRISMA checklist for systematic reviews; S2: Detailed search strings used in each database; S3: List of studies included in the systematic review (*n* = 52); S4: List of studies excluded after full text evaluation (*n* = 35).

## References

[1] EFSA (2011). Scientific Opinion on the public health hazards to be covered by inspection of meat (swine). EFSA J..

[2] Baer A.A., Miller M.J., Dilger A.C. (2013). Pathogens of Interest to the Pork Industry: A Review of Research on Interventions to Assure Food Safety. Compr. Rev. Food Sci. Food Saf..

[3] Blagojevic B., Nesbakken T., Alvseike O., Vågsholm I., Antic D., Johler S., Houf K., Meemken D., Nastasijevic I., Pinto M.V. (2021). Drivers, opportunities, and challenges of the European risk-based meat safety assurance system. Food Control..

[4] Pessoa J., Rodrigues da Costa M., Nesbakken T., Meemken D., RIBMINS Cost Action (2021). Assessment of the Effectiveness of Pre-harvest Meat Safety Interventions to Control Foodborne Pathogens in Broilers: A Systematic Review. Curr. Clin. Microbiol. Rep..

[5] Moher D., Liberati A., Tetzlaff J., Altman D.G., PRISMA Group (2009). Preferred reporting items for systematic reviews and meta-analyses: The PRISMA statement. BMJ.

[6] EFSA (2010). Application of systematic review methodology to food and feed safety assessments to support decision making. EFSA J..

[7] Bratz K., Gölz G., Janczyk P., Nöckler K., Alter T. (2015). Analysis of in vitro and in vivo effects of probiotics against *Campylobacter* spp.. Berl. Munch. Tierarztl. Wochenschr..

[8] Hasan S., Saha S., Junnikkala S., Orro T., Peltoniemi O., Oliviero C. (2019). Late gestation diet supplementation of resin acid-enriched composition increases sow colostrum immunoglobulin G content, piglet colostrum intake and improve sow gut microbiota. Animal.

[9] Kelneric Z., Naglic T., Udovicic I. (1996). Prevention of necrotic enteritis in piglets by vaccination of pregnant gilts with a *Clostridium perfringens* type C and D bacterin-toxoid. Vet. Med..

[10] Hammer J.M., Fuhrman M., Walz M. (2008). Serological evaluation of a *Clostridium perfringens* type A toxoid in a commercial swine herd. J. Swine Health Prod..

[11] Oliveira C.A., Silva R.O.S., Lage A.P., Coura F.M., Ramos C.P., Alfieri A.A., Guedes R.M.C., Lobato F.C.F. (2019). Non-toxigenic strain of *Clostridioides difficile* Z31 reduces the occurrence of *C. difficile* infection (CDI) in one-day-old piglets on a commercial pig farm. Vet. Microbiol..

[12] Richard O.K., Grahofer A., Nathues H., Posthaus H. (2019). Vaccination against *Clostridium perfringens* type C enteritis in pigs: A field study using an adapted vaccination scheme. Porc. Health Manag..

[13] Unterweger C., Kahler A., Gerlach G.F., Viehmann M., von Altrock A., Hennig-Pauka I. (2017). Administration of non-pathogenic isolates of *Escherichia coli* and *Clostridium perfringens* type A to piglets in a herd affected with a high incidence of neonatal diarrhoea. Animal.

[14] Pletinckx L.J., Dewulf J., De Bleecker Y., Rasschaert G., Goddeeris B.M., De Man I. (2013). Effect of a disinfection strategy on the methicillin-resistant *Staphylococcus aureus* CC398 prevalence of sows, their piglets and the barn environment. J. Appl. Microbiol..

[15] Hines M.E., Frazier K.S., Baldwin C.A., Cole J.R., Sangster L.T. (1998). Efficacy of vaccination for *Mycobacterium avium* with whole cell and subunit vaccines in experimentally infected swine. Vet. Microbiol..

[16] Jones F.T., Langlois B.E., Cromwell G.L., Hays V.W. (1983). Effect of feeding chlortetracycline or virginiamycin on shedding of salmonellae from experimentally-infected swine. J. Anim. Sci..

[17] Dahl J., Wingstrand A., Nielsen B., Baggesen D.L. (1997). Elimination of *Salmonella* typhimurium infection by the strategic movement of pigs. Vet. Rec..

[18] Nietfeld J.C., Feder I., Kramer T.T., Schoneweis D., Chengappa M.M. (1998). Preventing *Salmonella* infection in pigs with offsite weaning. Swine Health Prod..

[19] Rajkowski K.T., Eblen S., Laubauch C. (1998). Efficacy of washing and sanitizing trailers used for swine transport in reduction of *Salmonella* and *Escherichia coli*. J. Food Prot..

[20] Isaacson R.E., Firkins L.D., Weigel R.M., Zuckermann F.A., DiPietro J.A. (1999). Effect of transportation and feed withdrawal on shedding of *Salmonella* Typhimurium among experimentally infected pigs. Am. J. Vet. Res..

[21] Maes D., Gibson K., Trigo E., Saszak A., Grass J., Carlson A., Blaha T. (2001). Evaluation of cross-protection afforded by a *Salmonella* Choleraesuis vaccine against *Salmonella* infections in pigs under field conditions. Berl. Munch. Tierarztl. Wochenschr..

[22] Van der Wolf P.J., van Schie F.W., Elbers A.R., Engel B., van der Heijden H.M., Hunneman W.A., Tielen M.J. (2001). Administration of acidified drinking water to finishing pigs in order to prevent *Salmonella* infections. Vet. Q..

[23] Van Winsen R.L., Keuzenkamp D., Urlings B.A.P., Lipman L.J.A., Snijders J.A.M., Verheijden J.H.M., van Knapen F. (2002). Effect of fermented feed on shedding of *Enterobacteriaceae* by fattening pigs. Vet. Microbiol..

[24] Roesler U., Vonaltrock A., Heller P., Bremerich S., Arnold T., Lehmann J., Waldmann K.H., Truyen U., Hensel A. (2005). Effects of fluorequinolone treatment acidified feed, and improved hygiene measures on the occurrence of *Salmonella* Typhimurium DT104 in an integrated pig breeding herd. J. Vet. Med. B Infect. Dis. Vet. Public Health.

[25] Roesler U., Heller P., Waldmann K.H., Truyen U., Hensel A. (2006). Immunization of sows in an integrated pig-breeding herd using a homologous inactivated *Salmonella* vaccine decreases the prevalence of *Salmonella* typhimurium infection in the offspring. J. Vet. Med. B Infect. Dis. Vet. Public Health.

[26] Creus E., Perez J.F., Peralta B., Baucells F., Mateu E. (2007). Effect of acidified feed on the prevalence of *Salmonella* in market-age pigs. Zoonoses Public Health.

[27] Funk J., Wittum T.E., LeJeune J.T., Rajala-Schultz P.J., Bowman A., Mack A. (2007). Evaluation of stocking density and subtherapeutic chlortetracycline on *Salmonella enterica* subsp. enterica shedding in growing swine. Vet. Microbiol..

[28] Patchanee P., Crenshaw T.D., Bahnson P.B. (2007). Oral sodium chlorate, topical disinfection, and younger weaning age *reduce Salmonella enterica* shedding in pigs. J. Food Prot..

[29] Mannion C., Egan J., Lynch B.P., Fanning S., Leonard N. (2008). An investigation into the efficacy of washing trucks following the transportation of pigs—A *Salmonella* perspective. Foodborne Pathog. Dis..

[30] De Busser E.V., Dewulf J., Nollet N., Houf K., Schwarzer K., De Sadeleer L., De Zutter L., Maes D. (2009). Effect of organic acids in drinking water during the last 2 weeks prior to slaughter on *Salmonella* shedding by slaughter pigs and contamination of carcasses. Zoonoses Public Health.

[31] Rostagno M.H., Hurd H.S., McKean J.D. (2009). Split marketing as a risk factor for *Salmonella enterica* infection in swine. Foodborne Pathog. Dis..

[32] Visscher C.F., Winter P., Verspohl J., Stratmann-Selke J., Upmann M., Beyerbach M., Kamphues J. (2009). Effects of feed particle size at dietary presence of added organic acids on caecal parameters and the prevalence of *Salmonella* in fattening pigs on farm and at slaughter. J. Anim. Physiol. Anim. Nutr..

[33] Farzan A., Friendship R.M. (2010). A clinical field trial to evaluate the efficacy of vaccination in controlling *Salmonella* infection and the association of *Salmonella*-shedding and weight gain in pigs. Can. J. Vet. Res. Rev. Can. Rech. Vet..

[34] Arguello H., Carvajal A., Costillas S., Rubio P. (2013). Effect of the Addition of Organic Acids in Drinking Water or Feed During Part of the Finishing Period on the Prevalence of *Salmonella* in Finishing Pigs. Foodborne Pathog. Dis..

[35] Arguello H., Carvajal A., Naharro G., Rubio P. (2013). Evaluation of protection conferred by a *Salmonella* Typhimurium inactivated vaccine in *Salmonella*-infected finishing pig farms. Comp. Immunol. Microbiol. Infect. Dis..

[36] De Ridder L., Maes D., Dewulf J., Pasmans F., Boyen F., Haesebrouck F., Meroc E., Roels S., Leyman B., Butaye P. (2013). Effect of a DIVA vaccine with and without in-feed use of coated calcium-butyrate on transmission of *Salmonella* Typhimurium in pigs. BMC Vet. Res..

[37] Foss D.L., Agin T.S., Bade D., Dearwester D.A., Jolie R., Keich R.L., Lohse R.M., Reed M., Rosey E.L., Schneider P.A. (2013). Protective immunity to *Salmonella enterica* is partially serogroup specific. Vet. Immunol. Immunopathol..

[38] De Ridder L., Maes D., Dewulf J., Butaye P., Pasmans F., Boyen F., Haesebrouck F., Van der Stede Y. (2014). Use of a live attenuated *Salmonella enterica* serovar Typhimurium vaccine on farrow-to-finish pig farms. Vet. J..

[39] Kim H.B., Singer R.S., Borewicz K., White B.A., Sreevatsan S., Johnson T.J., Espejo L.A., Isaacson R.E. (2014). Effects of tylosin administration on C-reactive protein concentration and carriage of *Salmonella enterica* in pigs. Am. J. Vet. Res..

[40] Stojanac N., Stevancevic O., Potkonjak A., Savic B., Stancic I., Vracar V. (2014). The impact of space allowance on productivity performance and *Salmonella* spp. shedding in nursery pigs. Livestig. Sci..

[41] Yin F.G., Farzan A., Wang Q., Yu H., Yin Y.L., Hou Y.Q., Friendship R., Gong J.S. (2014). Reduction of *Salmonella enterica* Serovar Typhimurium DT104 Infection in Experimentally Challenged Weaned Pigs Fed a Lactobacillus-Fermented Feed. Foodborne Pathog. Dis..

[42] Artuso-Ponte V., Moeller S., Rajala-Schultz P., Medardus J.J., Munyalo J., Lim K., Gebreyes W.A. (2015). Supplementation with Quaternary Benzo(c) phenanthridine Alkaloids Decreased Salivary Cortisol and *Salmonella* Shedding in Pigs After Transportation to the Slaughterhouse. Foodborne Pathog. Dis..

[43] Grilli E., Foresti F., Tugnoli B., Fustini M., Zanoni M.G., Pasquali P., Callaway T.R., Piva A., Alborali G.L. (2015). Microencapsulated Sorbic Acid and Pure Botanicals Affect *Salmonella* Typhimurium Shedding in Pigs: A Close-Up Look from Weaning to Slaughter in Controlled and Field Conditions. Foodborne Pathog. Dis..

[44] Bearson B.L., Bearson S.M.D., Kich J.D. (2016). A DIVA vaccine for cross-protection against *Salmonella*. Vaccine.

[45] Rasschaert G., Michiels J., Tagliabue M., Missotten J., De Smet S., Heyndrickx M. (2016). Effect of Organic Acids on *Salmonella* Shedding and Colonization in Pigs on a Farm with High *Salmonella* Prevalence. J. Food Prot..

[46] Walia K., Argüello H., Lynch H., Leonard F.C., Grant J., Yearsley D., Kelly S., Duffy G., Gardiner G.E., Lawlor P.G. (2016). Effect of feeding sodium butyrate in the late finishing period on *Salmonella* carriage, seroprevalence, and growth of finishing pigs. Prev. Vet. Med..

[47] Casanova-Higes A., Andres-Barranco S., Mainar-Jaime R.C. (2017). Effect of the addition of protected sodium butyrate to the feed on *Salmonella* spp. infection dynamics in fattening pigs. Anim. Feed Sci. Technol..

[48] Eicher S.D., Rostagno M.H., Lay D.C. (2017). Feed withdrawal and transportation effects on *Salmonella enterica* levels in market-weight pigs. J. Anim. Sci..

[49] Lynch H., Leonard F.C., Walla K., Lawlor P.G., Duffy G., Fanning S., Markey B.K., Brady C., Gardiner G.E., Arguello H. (2017). Investigation of in-feed organic acids as a low cost strategy to combat *Salmonella* in grower pigs. Prev. Vet. Med..

[50] Martelli F., Lambert M., Butt P., Cheney T., Tatone F.A., Callaby R., Rabie A., Gosling R.J., Fordon S., Crocker G. (2017). Evaluation of an enhanced cleaning and disinfection protocol in *Salmonella* contaminated pig holdings in the United Kingdom. PLoS ONE.

[51] Walia K., Arguello H., Lynch H., Leonard F.C., Grant J., Yearsley D., Kelly S., Duffy G., Gardiner G.E., Lawlor P.G. (2017). Effect of strategic administration of an encapsulated blend of formic acid, citric acid, and essential oils on *Salmonella* carriage, seroprevalence, and growth of finishing pigs. Prev. Vet. Med..

[52] Casanova-Higes A., Andres-Barranco S., Mainar-Jaime R.C. (2018). Use of a new form of protected sodium butyrate to control *Salmonella* infection in fattening pigs. Span. J. Agric. Res..

[53] Leite F.L.L., Singer R.S., Ward T., Gebhart C.J., Isaacson R.E. (2018). Vaccination Against Lawsonia intracellularis Decreases Shedding of *Salmonella enterica* serovar Typhimurium in Co-Infected Pigs and Alters the Gut Microbiome. Sci Rep..

[54] Smith R.P., Andres V., Martelli F., Gosling B., Marco-Jimenez F., Vaughan K., Tchorzewska M., Davies R. (2018). Maternal vaccination as a *Salmonella* Typhimurium reduction strategy on pig farms. J. Appl. Microbiol..

[55] Holman D.B., Bearson B.L., Allen H.K., Shippy D.C., Loving C.L., Kerr B.J., Bearson S.M.D., Brunelle B.W. (2019). Chlortetracycline Enhances Tonsil Colonization and Fecal Shedding of Multidrug-Resistant *Salmonella enterica* Serovar Typhimurium DT104 without Major Alterations to the Porcine Tonsillar and Intestinal Microbiota. Appl. Environ. Microbiol..

[56] Peeters L., Dewulf J., Boyen F., Brossé C., Vandersmissen T., Rasschaert G., Heyndrickx M., Cargnel M., Pasmans F., Maes D. (2019). Effects of attenuated vaccine protocols against *Salmonella* Typhimurium on *Salmonella* serology in subclinically infected pig herds. Vet. J..

[57] Costa E.D., Kich J.D., Miele M., Mores N., Amaral A., Coldebella A., Cardoso M., Corbellini L.G. (2020). Evaluation of two strategies for reducing the spread of *Salmonella* in commercial swine herds during the finishing phase and their incremental cost-effectiveness ratios. Semin. Cienc. Agrar..

[58] Peeters L., Dewulf J., Boyen F., Brosse C., Vandersmissen T., Rasschaert G., Heyndrickx M., Cargnel M., Mattheus W., Pasmans F. (2020). Bacteriological evaluation of vaccination against *Salmonella* Typhimurium with an attenuated vaccine in subclinically infected pig herds. Prev. Vet. Med..

[59] European Food Safety Authority EFSA, European Centre for Disease Prevention and Control ECDC (2021). The European Union One Health 2019 Zoonoses Report. EFSA J..

[60] Davies P.R., Scott Hurd H., Funk J.A., Fedorka-Cray P.J., Jones F.T. (2004). The role of contaminated feed in the epidemiology and control of *Salmonella enterica* in pork production. Foodborne Pathog. Dis..

[61] Nesbakken T., Skjerve E., Lium B. The succesful control of *Salmonella* in Norway. Proceedings of the 13th Safepork.

[62] De la Cruz M.L., Conrado I., Nault A., Perez A., Dominguez L., Alvarez J. (2017). Vaccination as a control strategy against *Salmonella* infection in pigs: A systematic review and meta-analysis of the literature. Res. Vet. Sci..

[63] Wilhelm B., Rajić A., Parker S., Waddell L., Sanchez J., Fazil A., Wilkins W., McEwen S.A. (2012). Assessment of the efficacy and quality of evidence for five on-farm interventions for *Salmonella* reduction in grow-finish swine: A systematic review and meta-analysis. Prev. Vet. Med..

[64] Wilhelm B.J., Young I., Cahill S., Nakagawa R., Desmarchelier P., Rajić A. (2017). Rapid systematic review and meta-analysis of the evidence for effectiveness of primary production interventions to control *Salmonella* in beef and pork. Prev. Vet. Med..

[65] Weijtens M.J., van der Plas J., Bijker P.G., Urlings H.A., Koster D., van Logtestijn J.G., Huis in’t Veld J.H. (1997). The transmission of *campylobacter* in piggeries; an epidemiological study. J. Appl. Microbiol..

[66] Young C.R., Harvey R., Anderson R., Nisbet D., Stanker L.H. (2000). Enteric colonisation following natural exposure to Campylobacter in pigs. Res. Vet. Sci..

[67] Humphrey T., O’Brien S., Madsen M. (2007). *Campylobacters* as zoonotic pathogens: A food production perspective. Int. J. Food Microbiol..

[68] Nesbakken T., Eckner K., Rotterud O.J. (2008). The effect of blast chilling on occurrence of human pathogenic *Yersinia enterocolitica* compared to *Campylobacter* spp. and numbers of hygienic indicators on pig carcasses. Int. J. Food Microbiol..

[69] Chang V.P., Mills E.W., Cutter C.N. (2003). Reduction of bacteria on pork carcasses associated with chilling method. J. Food Prot..

[70] Kapperud G., Skjerve E., Bean N.H., Ostroff S.M., Lassen J. (1992). Risk factors for sporadic *Campylobacter* infections: Results of a case-control study in southeastern Norway. J. Clin. Microbiol..

[71] Kapperud G., Espeland G., Wahl E., Walde A., Herikstad H., Gustavsen S., Tveit I., Natas O., Bevanger L., Digranes A. (2003). Factors associated with increased and decreased risk of *Campylobacter* infection: A prospective case-control study in Norway. Am. J. Epidemiol..

[72] Roux F., Sproston E., Rotariu O., Macrae M., Sheppard S.K., Bessell P., Smith-Palmer A., Cowden J., Maiden M.C., Forbes K.J. (2013). Elucidating the aetiology of human *Campylobacter coli* infections. PLoS ONE.

[73] Elstrom P., Grontvedt C.A., Gabrielsen C., Stegger M., Angen O., Amdal S., Enger H., Urdahl A.M., Jore S., Steinbakk M. (2019). Livestock-Associated MRSA CC1 in Norway; Introduction to Pig Farms, Zoonotic Transmission, and Eradication. Front. Microbiol..

[74] Urdahl A.M., Norström M., Welde H., Bergsjø B., Grøntvedt C.A. (2020). The Surveillance Programme for Methicillin Resistant Staphylococcus aureus in Pigs in Norway 2019.

[75] Meester M., Tobias T.J., Bouwknegt M., Kusters N.E., Stegeman J.A., van der Poel W.H.M. (2021). Infection dynamics and persistence of hepatitis E virus on pig farms—A review. Porc. Health Manag..

[76] Krog J.S., Larsen L.E., Breum S.Ø. (2019). Tracing Hepatitis E Virus in Pigs From Birth to Slaughter. Front. Vet. Sci..

[77] Skjerve E., Lium B., Nielsen B., Nesbakken T. (1998). Control of *Yersinia enterocolitica* in pigs at herd level. Int. J. Food Microbiol..

[78] Virtanen S., Salonen L., Laukkanen-Ninios R., Fredriksson-Ahomaa M., Korkeala H. (2012). Piglets are a source of pathogenic *Yersinia enterocolitica* on fattening-pig farms. Appl. Env. Microbiol..

[79] Vilar M.J., Virtanen S., Heinonen M., Korkeala H. (2013). Management practices associated with the carriage of *Yersinia enterocolitica* in pigs at farm level. Foodborne Pathog. Dis..

[80] Virtanen S., Nikunen S., Korkeala H. (2014). Introduction of infected animals to herds is an important route for the spread *of Yersinia enterocolitica* infection between pig farms. J. Food Prot..

[81] Nesbakken T., Iversen T., Lium B. (2007). Pig herds free from human pathogenic *Yersinia enterocolitica*. Emerg. Infect. Dis.

[82] Kolstoe E.M., Iversen T., Ostensvik O., Abdelghani A., Secic I., Nesbakken T. (2015). Specific pathogen-free pig herds also free from *Campylobacter*?. Zoonoses Public Health.

[83] Nesbakken T., Sofos J. (2015). 2—Update on *Yersinia* as a foodborne pathogen: Analysis and control. Advances in Microbial Food Safety.

[84] Laukkanen-Ninios R., Fredriksson-Ahomaa M., Korkeala H. (2014). Enteropathogenic *Yersinia* in the Pork Production Chain: Challenges for Control. Compr. Rev. Food Sci. Food Saf..

[85] Tenter A.M., Heckeroth A.R., Weiss L.M. (2000). *Toxoplasma gondii*: From animals to humans. Int. J. Parasitol..

[86] Skjerve E., Tharaldsen J., Waldeland H., Kapperud G., Nesbakken T. (1996). Antibodies to *Toxoplasma gondii* in Norwegian slaughtered sheep, pigs and cattle. Bull. Scand. Soc. Parasitol..

[87] Gebreyes W.A., Bahnson P.B., Funk J.A., McKean J., Patchanee P. (2008). Seroprevalence of *Trichinella*, *Toxoplasma*, and *Salmonella* in antimicrobial-free and conventional swine production systems. Foodborne Pathog. Dis..

[88] Mateus-Pinilla N.E., Dubey J.P., Choromanski L., Weigel R.M. (1999). A field trial of the effectiveness of a feline *Toxoplasma gondii* vaccine in reducing *T-gondii* exposure for swine. J. Parasitol..

[89] Ortega-Pacheco A., Acosta-Viana K.Y., Guzman-Marin E., Uitzil-Alvarez B., Rodriguez-Buenfil J.C., Jimenez-Coello M. (2011). Infection dynamic of *Toxoplasma gondii* in two fattening pig farms exposed to high and low cat density in an endemic region. Vet. Parasitol..

