# Supplementary material for: A Systematic Review on the Effectiveness of Pre-Harvest Meat Safety Interventions in Pig Herds to Control Salmonella and Other Foodborne Pathogens
Source: Microorganisms. 2021 Aug 27;9(9):1825. doi: 10.3390/microorganisms9091825 (PMC8466550; doi:10.3390/microorganisms9091825)
Supplement: Supplementary file 1 [file microorganisms-09-01825-s001.zip › S2 Detailed search strings used_title corrected.pdf]

S1: Detailed search strings used in each database

The yellow string should be replaced by each of the 15 pathogenic agents included in this systematic review. The corresponded search strings for those are included in Table 1 as "Keyword and/or string searched".

PubMed:

(pig*[Title/Abstract] OR swine[Title/Abstract] OR sow[Title/Abstract] OR porcine[Title/Abstract] OR hog[Title/Abstract]) AND (KEYWORDS)[Title/Abstract] AND (on-farm[Title/Abstract] OR on farm[Title/Abstract] OR pre-harvest[Title/Abstract] OR herd[Title/Abstract] OR transport[Title/Abstract] OR movement)[Title/Abstract] AND (biosecurity[Title/Abstract] OR biocontainment[Title/Abstract] OR vaccin*[Title/Abstract] OR clean*[Title/Abstract] OR disinfect*[Title/Abstract] OR wash*[Title/Abstract] OR antibiotic[Title/Abstract] OR antimicrobial*[Title/Abstract] OR anti-parasitic*[Title/Abstract] OR deworm*[Title/Abstract] OR worm* anti-helmint*[Title/Abstract] OR feed*[Title/Abstract] OR feed* strateg*[Title/Abstract] OR feed* practic*[Title/Abstract] OR feed form[Title/Abstract] OR particle size[Title/Abstract] OR water[Title/Abstract] OR acid*[Title/Abstract] OR feed* additive*[Title/Abstract] OR feed* supplement*[Title/Abstract] OR eradicat*[Title/Abstract] OR eliminat*[Title/Abstract] OR control*[Title/Abstract] OR hygien*[Title/Abstract] OR manage*[Title/Abstract] OR husbandry)[Title/Abstract]

Web of knowledge:

1. *Title searches*

(TI=((pig* OR swine OR sow OR porcine OR hog) AND KEYWORDS AND (on-farm OR on farm OR pre-harvest OR herd OR transport OR movement) AND (biosecurity OR biocontainment OR vaccin* OR clean* OR disinfect* OR wash* OR antibiotic OR antimicrobial* OR anti-parasitic* OR deworm* OR worm* anti-helmint* OR feed* OR feed* strateg* OR feed* practic* OR feed form OR particle size OR water OR acid* OR feed* additive* OR feed* supplement* OR eradicat* OR eliminat* OR control* OR hygien* OR manage* OR husbandry)))

2. *Abstract searches*

(AB=((pig* OR swine OR sow OR porcine OR hog) AND KEYWORDS AND (on-farm OR on farm OR pre-harvest OR herd OR transport OR movement) AND (biosecurity OR biocontainment OR vaccin* OR clean* OR disinfect* OR wash* OR antibiotic OR antimicrobial* OR anti-parasitic* OR deworm* OR worm* anti-helmint* OR feed* OR feed* strateg* OR feed* practic* OR feed form OR particle size OR water OR acid* OR feed* additive* OR feed* supplement* OR eradicat* OR eliminat* OR control* OR hygien* OR manage* OR husbandry)))
