# Supplementary material for: A Systematic Review on the Effectiveness of Pre-Harvest Meat Safety Interventions in Pig Herds to Control Salmonella and Other Foodborne Pathogens
Source: Microorganisms. 2021 Aug 27;9(9):1825. doi: 10.3390/microorganisms9091825 (PMC8466550; doi:10.3390/microorganisms9091825)
Supplement: Supplementary file 1 [file microorganisms-09-01825-s001.zip › S3 list of studies included_title corrected.pdf]

S2: List of studies included in the systematic review (n=52)

Arguello, H., Carvajal, A., Costillas, S., & Rubio, P. (2013a). Effect of the Addition of Organic Acids in Drinking Water or Feed During Part of the Finishing Period on the Prevalence of Salmonella in Finishing Pigs. *Foodborne Pathogens and Disease, 10*(10), 842-849. doi:10.1089/fpd.2013.1497

Arguello, H., Carvajal, A., Naharro, G., & Rubio, P. (2013b). Evaluation of protection conferred by a Salmonella Typhimurium inactivated vaccine in Salmonella-infected finishing pig farms. *Comparative Immunology Microbiology and Infectious Diseases, 36*(5), 489-498. doi:10.1016/j.cimid.2013.05.002

Artuso-Ponte, V., Moeller, S., Rajala-Schultz, P., Medardus, J. J., Munyalo, J., Lim, K., & Gebreyes, W. A. (2015). Supplementation with Quaternary Benzo(c) phenanthridine Alkaloids Decreased Salivary Cortisol and Salmonella Shedding in Pigs After Transportation to the Slaughterhouse. *Foodborne Pathogens and Disease, 12*(11), 891-897. doi:10.1089/fpd.2015.2009

Bearson, B. L., Bearson, S. M. D., & Kich, J. D. (2016). A DIVA vaccine for cross-protection against Salmonella. *Vaccine, 34*(10), 1241-1246. doi:10.1016/j.vaccine.2016.01.036

Bratz, K., Gölz, G., Janczyk, P., Nöckler, K., & Alter, T. (2015). Analysis of in vitro and in vivo effects of probiotics against Campylobacter spp. *Berl Munch Tierarztl Wochenschr, 128*(3-4), 155-162.

Casanova-Higes, A., Andres-Barranco, S., & Mainar-Jaime, R. C. (2017). Effect of the addition of protected sodium butyrate to the feed on Salmonella spp. infection dynamics in fattening pigs. *Animal Feed Science and Technology, 231*, 12-18. doi:10.1016/j.anifeedsci.2017.06.008

Casanova-Higes, A., Andres-Barranco, S., & Mainar-Jaime, R. C. (2018). Use of a new form of protected sodium butyrate to control Salmonella infection in fattening pigs. *Spanish Journal of Agricultural Research, 16*(4), 5. doi:10.5424/sjar/2018164-13888

Costa, E. D., Kich, J. D., Miele, M., Mores, N., Amaral, A., Coldebella, A., . . . Corbellini, L. G. (2020). Evaluation of two strategies for reducing the spread of Salmonella in commercial swine herds during the finishing phase and their incremental cost-effectiveness ratios. *Semina-Ciencias Agrarias, 41*(2), 505-516. doi:10.5433/1679-0359.2020v41n2p505

Creus, E., Perez, J. F., Peralta, B., Baucells, F., & Mateu, E. (2007). Effect of acidified feed on the prevalence of Salmonella in market-age pigs. *Zoonoses and Public Health, 54*(8), 314-319. doi:10.1111/j.1863-2378.2007.01069.x

Dahl, J., Wingstrand, A., Nielsen, B., & Baggesen, D. L. (1997). Elimination of Salmonella typhimurium infection by the strategic movement of pigs. *Veterinary Record, 140*(26), 679-681. doi:10.1136/vr.140.26.679

De Busser, E. V., Dewulf, J., Nollet, N., Houf, K., Schwarzer, K., De Sadeleer, L., . . . Maes, D. (2009). Effect of organic acids in drinking water during the last 2 weeks prior to slaughter on Salmonella shedding by slaughter pigs and contamination of carcasses. *Zoonoses Public Health, 56*(3), 129-136. doi:10.1111/j.1863-2378.2008.01172.x

De Ridder, L., Maes, D., Dewulf, J., Butaye, P., Pasmans, F., Boyen, F., . . . Van der Stede, Y. (2014). Use of a live attenuated Salmonella enterica serovar Typhimurium vaccine on farrow-to-finish pig farms. *Vet J, 202*(2), 303-308. doi:10.1016/j.tvjl.2014.09.012

De Ridder, L., Maes, D., Dewulf, J., Pasmans, F., Boyen, F., Haesebrouck, F., . . . Van der Stede, Y. (2013). Effect of a DIVA vaccine with and without in-feed use of coated calcium-butyrate on transmission of Salmonella Typhimurium in pigs. *Bmc Veterinary Research, 9*, 8. doi:10.1186/1746-6148-9-243

Eicher, S. D., Rostagno, M. H., & Lay, D. C. (2017). Feed withdrawal and transportation effects on Salmonella enterica levels in market-weight pigs. *J Anim Sci, 95*(7), 2848-2858. doi:10.2527/jas.2017.1454

Farzan, A., & Friendship, R. M. (2010). A clinical field trial to evaluate the efficacy of vaccination in controlling Salmonella infection and the association of Salmonella-shedding and weight gain in pigs. *Canadian Journal of Veterinary Research-Revue Canadienne De Recherche Veterinaire, 74*(4), 258-263.

Foss, D. L., Agin, T. S., Bade, D., Dearwester, D. A., Jolie, R., Keich, R. L., . . . Willy, M. S. (2013). Protective immunity to Salmonella enterica is partially serogroup specific. *Vet Immunol Immunopathol, 155*(1-2), 76-86. doi:10.1016/j.vetimm.2013.06.007

Funk, J., Wittum, T. E., LeJeune, J. T., Rajala-Schultz, P. J., Bowman, A., & Mack, A. (2007). Evaluation of stocking density and subtherapeutic chlortetracycline on Salmonella enterica subsp. enterica shedding in growing swine. *Vet Microbiol, 124*(3-4), 202-208. doi:10.1016/j.vetmic.2007.04.018

Grilli, E., Foresti, F., Tugnoli, B., Fustini, M., Zanoni, M. G., Pasquali, P., . . . Alborali, G. L. (2015). Microencapsulated Sorbic Acid and Pure Botanicals Affect Salmonella Typhimurium Shedding in Pigs: A Close-Up Look from Weaning to Slaughter in Controlled and Field Conditions. *Foodborne Pathogens and Disease, 12*(10), 813-819. doi:10.1089/fpd.2015.1953

Hammer, J. M., Fuhrman, M., & Walz, M. (2008). Serological evaluation of a Clostridium perftingens type A toxoid in a commercial swine herd. *Journal of Swine Health and Production, 16*(1), 37-40.

Hasan, S., Saha, S., Junnikkala, S., Orro, T., Peltoniemi, O., & Oliviero, C. (2019). Late gestation diet supplementation of resin acid-enriched composition increases sow colostrum immunoglobulin G content, piglet colostrum intake and improve sow gut microbiota. *Animal, 13*(8), 1599-1606. doi:10.1017/s1751731118003518

Hines, M. E., 2nd, Frazier, K. S., Baldwin, C. A., Cole, J. R., Jr., & Sangster, L. T. (1998). Efficacy of vaccination for Mycobacterium avium with whole cell and subunit vaccines in experimentally infected swine. *Vet Microbiol, 63*(1), 49-59. doi:10.1016/s0378-1135(98)00224-7

Holman, D. B., Bearson, B. L., Allen, H. K., Shippy, D. C., Loving, C. L., Kerr, B. J., . . . Brunelle, B. W. (2019). Chlortetracycline Enhances Tonsil Colonization and Fecal Shedding of Multidrug-Resistant Salmonella enterica Serovar Typhimurium DT104 without Major Alterations to the Porcine Tonsillar and Intestinal Microbiota. *Applied and Environmental Microbiology, 85*(4), 12. doi:10.1128/aem.02354.18

Isaacson, R. E., Firkins, L. D., Weigel, R. M., Zuckermann, F. A., & DiPietro, J. A. (1999). Effect of transportation and feed withdrawal on shedding of Salmonella Typhimurium among experimentally infected pigs. *American Journal of Veterinary Research, 60*(9), 1155-1158.

Jones, F. T., Langlois, B. E., Cromwell, G. L., & Hays, V. W. (1983). Effect of feeding chlortetracycline or virginiamycin on shedding of salmonellae from experimentally-infected swine. *J Anim Sci, 57*(2), 279-285. doi:10.2527/jas1983.572279x

Kelneric, Z., Naglic, T., & Udovicic, I. (1996). Prevention of necrotic enteritis in piglets by vaccination of pregnant gilts with a Clostridium perfringens type C and D bacterin-toxoid. *Veterinarni Medicina, 41*(11), 335-338.

Kim, H. B., Singer, R. S., Borewicz, K., White, B. A., Sreevatsan, S., Johnson, T. J., . . . Isaacson, R. E. (2014). Effects of tylosin administration on C-reactive protein concentration and carriage of Salmonella enterica in pigs. *American Journal of Veterinary Research, 75*(5), 460-467. doi:10.2460/ajvr.75.5.460

Leite, F. L. L., Singer, R. S., Ward, T., Gebhart, C. J., & Isaacson, R. E. (2018). Vaccination Against Lawsonia intracellularis Decreases Shedding of Salmonella enterica serovar

Typhimurium in Co-Infected Pigs and Alters the Gut Microbiome. *Scientific Reports, 8*, 10. doi:10.1038/s41598-018-21255-7

Lynch, H., Leonard, F. C., Walla, K., Lawlor, P. G., Duffy, G., Fanning, S., . . . Arguello, H. (2017). Investigation of in-feed organic acids as a low cost strategy to combat Salmonella in grower pigs. *Preventive Veterinary Medicine, 139*, 50-57. doi:10.1016/j.prevetmed.2017.02.008

Maes, D., Gibson, K., Trigo, E., Saszak, A., Grass, J., Carlson, A., & Blaha, T. (2001). Evaluation of cross-protection afforded by a Salmonella Choleraesuis vaccine against Salmonella infections in pigs under field conditions. *Berl Munch Tierarztl Wochenschr, 114*(9-10), 339-341.

Mannion, C., Egan, J., Lynch, B. P., Fanning, S., & Leonard, N. (2008). An investigation into the efficacy of washing trucks following the transportation of pigs - A Salmonella perspective. *Foodborne Pathogens and Disease, 5*(3), 261-271. doi:10.1089/fpd.2007.0069

Martelli, F., Lambert, M., Butt, P., Cheney, T., Tatone, F. A., Callaby, R., . . . Smith, R. P. (2017). Evaluation of an enhanced cleaning and disinfection protocol in Salmonella contaminated pig holdings in the United Kingdom. *PLoS One, 12*(6), 20. doi:10.1371/journal.pone.0178897

Nietfeld, J. C., Feder, I., Kramer, T. T., Schoneweis, D., & Chengappa, M. M. (1998). Preventing Salmonella infection in pigs with offsite weaning. *Swine Health and Production, 6*(1), 27-32.

Oliveira, C. A., Silva, R. O. S., Lage, A. P., Coura, F. M., Ramos, C. P., Alfieri, A. A., . . . Lobato, F. C. F. (2019). Non-toxigenic strain of Clostridioides difficile Z31 reduces the occurrence of C. difficile infection (CDI) in one-day-old piglets on a commercial pig farm. *Veterinary Microbiology, 231*, 1-6. doi:10.1016/j.vetmic.2019.02.026

Patchanee, P., Crenshaw, T. D., & Bahnson, P. B. (2007). Oral sodium chlorate, topical disinfection, and younger weaning age reduce Salmonella enterica shedding in pigs. *Journal of Food Protection, 70*(8), 1798-1803. doi:10.4315/0362-028x-70.8.1798

Peeters, L., Dewulf, J., Boyen, F., Brosse, C., Vandersmissen, T., Rasschaert, G., . . . Maes, D. (2020). Bacteriological evaluation of vaccination against Salmonella Typhimurium with an attenuated vaccine in subclinically infected pig herds. *Preventive Veterinary Medicine, 182*, 11. doi:10.1016/j.prevetmed.2019.04.016

Peeters, L., Dewulf, J., Boyen, F., Brossé, C., Vandersmissen, T., Rasschaert, G., . . . Maes, D. (2019). Effects of attenuated vaccine protocols against Salmonella Typhimurium on Salmonella serology in subclinically infected pig herds. *Vet J, 249*, 67-72. doi:10.1016/j.tvjl.2019.05.008

Pletinckx, L. J., Dewulf, J., De Bleecker, Y., Rasschaert, G., Goddeeris, B. M., & De Man, I. (2013). Effect of a disinfection strategy on the methicillin-resistant Staphylococcus aureus CC398 prevalence of sows, their piglets and the barn environment. *Journal of Applied Microbiology, 114*(6), 1634-1641. doi:10.1111/jam.12201

Rajkowski, K. T., Eblen, S., & Laubauch, C. (1998). Efficacy of washing and sanitizing trailers used for swine transport in reduction of Salmonella and Escherichia coli. *J Food Prot, 61*(1), 31-35. doi:10.4315/0362-028x-61.1.31

Rasschaert, G., Michiels, J., Tagliabue, M., Missotten, J., De Smet, S., & Heyndrickx, M. (2016). Effect of Organic Acids on Salmonella Shedding and Colonization in Pigs on a Farm with High Salmonella Prevalence. *Journal of Food Protection, 79*(1), 51-58. doi:10.4315/0362-028x.Jfp-15-183

Richard, O. K., Grahofer, A., Nathues, H., & Posthaus, H. (2019). Vaccination against Clostridium perfringens type C enteritis in pigs: a field study using an adapted vaccination scheme. *Porcine Health Management, 5*(1), 9. doi:10.1186/s40813-019-0127-8

Roesler, U., Heller, P., Waldmann, K. H., Truyen, U., & Hensel, A. (2006). Immunization of sows in an integrated pig-breeding herd using a homologous inactivated Salmonella vaccine decreases the prevalence of Salmonella typhimurium infection in the offspring. *J Vet Med B Infect Dis Vet Public Health, 53*(5), 224-228. doi:10.1111/j.1439-0450.2006.00951.x

Roesler, U., Vonaltrock, A., Heller, P., Bremerich, S., Arnold, T., Lehmann, J., . . . Hensel, A. (2005). Effects of fluorequinolone treatment acidified feed, and improved hygiene measures on the occurrence of Salmonella Typhimurium DT104 in an integrated pig breeding herd. *J Vet Med B Infect Dis Vet Public Health, 52*(2), 69-74. doi:10.1111/j.1439-0450.2005.00825.x

Rostagno, M. H., Hurd, H. S., & McKean, J. D. (2009). Split marketing as a risk factor for Salmonella enterica infection in swine. *Foodborne Pathog Dis, 6*(7), 865-869. doi:10.1089/fpd.2009.0309

Smith, R. P., Andres, V., Martelli, F., Gosling, B., Marco-Jimenez, F., Vaughan, K., . . . Davies, R. (2018). Maternal vaccination as a Salmonella Typhimurium reduction strategy on pig farms. *Journal of Applied Microbiology, 124*(1), 274-285. doi:10.1111/jam.13609

Stojanac, N., Stevancevic, O., Potkonjak, A., Savic, B., Stancic, I., & Vracar, V. (2014). The impact of space allowance on productivity performance and Salmonella spp. shedding in nursery pigs. *Livestock Science, 164*, 149-153. doi:10.1016/j.livsci.2014.03.027

Unterweger, C., Kahler, A., Gerlach, G. F., Viehmann, M., von Altrock, A., & Hennig-Pauka, I. (2017). Administration of non-pathogenic isolates of Escherichia coli and Clostridium perfringens type A to piglets in a herd affected with a high incidence of neonatal diarrhoea. *Animal, 11*(4), 670-676. doi:10.1017/s1751731116001804

van der Wolf, P. J., van Schie, F. W., Elbers, A. R., Engel, B., van der Heijden, H. M., Hunneman, W. A., & Tielen, M. J. (2001). Administration of acidified drinking water to finishing pigs in order to prevent Salmonella infections. *Vet Q, 23*(3), 121-125. doi:10.1080/01652176.2001.9695097

van Winsen, R. L., Keuzenkamp, D., Urlings, B. A. P., Lipman, L. J. A., Snijders, J. A. M., Verheijden, J. H. M., & van Knapen, F. (2002). Effect of fermented feed on shedding of Enterobacteriaceae by fattening pigs. *Veterinary Microbiology, 87*(3), 267-276. doi:10.1016/s0378-1135(02)00066-4

Visscher, C. F., Winter, P., Verspohl, J., Stratmann-Selke, J., Upmann, M., Beyerbach, M., & Kamphues, J. (2009). Effects of feed particle size at dietary presence of added organic acids on caecal parameters and the prevalence of Salmonella in fattening pigs on farm and at slaughter. *Journal of Animal Physiology and Animal Nutrition, 93*(4), 423-430. doi:10.1111/j.1439-0396.2008.00821.x

Walia, K., Arguello, H., Lynch, H., Leonard, F. C., Grant, J., Yearsley, D., . . . Lawlor, P. G. (2017). Effect of strategic administration of an encapsulated blend of formic acid, citric acid, and essential oils on Salmonella carriage, seroprevalence, and growth of finishing pigs. *Preventive Veterinary Medicine, 137*, 28-35. doi:10.1016/j.prevetmed.2016.12.007

Walia, K., Argüello, H., Lynch, H., Leonard, F. C., Grant, J., Yearsley, D., . . . Lawlor, P. G. (2016). Effect of feeding sodium butyrate in the late finishing period on Salmonella carriage, seroprevalence, and growth of finishing pigs. *Prev Vet Med, 131*, 79-86. doi:10.1016/j.prevetmed.2016.07.009

Yin, F. G., Farzan, A., Wang, Q., Yu, H., Yin, Y. L., Hou, Y. Q., . . . Gong, J. S. (2014). Reduction of Salmonella enterica Serovar Typhimurium DT104 Infection in Experimentally Challenged Weaned Pigs Fed a Lactobacillus-Fermented Feed. *Foodborne Pathogens and Disease, 11*(8), 628-634. doi:10.1089/fpd.2013.1676
